# Psoas Hydatid Cyst in Children: A Rare Localization about a Case

**DOI:** 10.1155/2021/1961509

**Published:** 2021-11-06

**Authors:** Amine El Khassoui, El Mouhtadi Aghoutane, Tarik Salama, Redouane El Fezzazi

**Affiliations:** Traumatology and Orthopedic Pediatric Surgery Department, University Hospital of Marrakech, Marrakech, Morocco

## Abstract

**Introduction:**

The development of hydatid cysts in the muscle is rare, and it is even rarer in children. We report the case of a 9-year-old child treated in pediatric orthopedics department at the University Hospital of Marrakech for a hydatid cyst psoas muscle revealed by lameness.

**Result:**

The child was consulted for painless and afebrile lameness of the left hip evolving since 3 months. The clinical examination finds a mass of the left flank. Investigations based on the abdominal ultrasound in first intention showed a hydatid cyst depending on the left psoas muscle. Pelvic CT and abdominal MRI were able to confirm the diagnosis and allowed a better study of the cyst neighboring elements. The drainage of the cyst followed by pericystectomy after evacuation of the vesicles contained in the cyst was done as radical treatment.

**Conclusion:**

The hydatid cyst of the psoas is a rare entity in the child requiring a good radiological study of the cyst as well as its neighboring elements to propose the most adapted surgery.

## 1. Introduction

Human is an accidental host in the parasitic cycle of *Taenia Echinococcus granulosus* responsible for cystic formation called hydatid disease or echinococciasis. Hydatid cyst is still a public health problem in several countries where it is endemic. The muscle is an uncommon location for hydatid cyst, accounting for only 2 to 3% of cases [1]. We report a case of an unusual localization hydatid cyst in the psoas muscle in a 9-year-old child.

## 2. Observation

**Figure 1:**
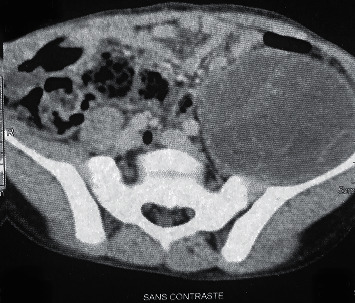
CT scan showing a well-limited hydatid cyst in the expansion of left psoas muscle.

**Figure 2:**
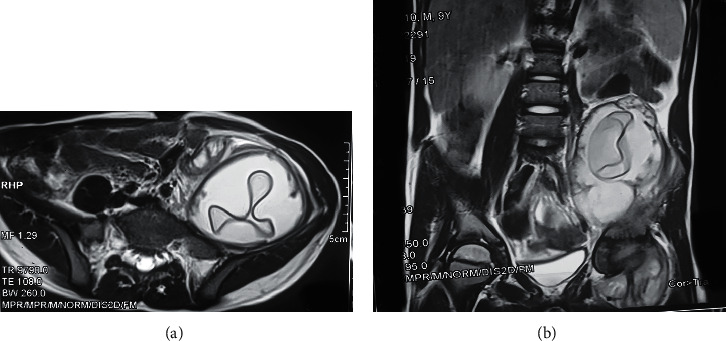
MRI image in transversal (a) and sagittal (b) plane showing a floating membrane of the hydatid cyst.

We report the case of a 9-year-old boy admitted to the pediatric emergency unit for painless limp without fever since 3 months. He had no alterations in his objective examination upon admission. He had no significant previous medical events, but alluded to having regular contact with dogs and sheep in Douar where he lived in the rural zone of Kalaa Sraghna city in Morocco. The physical examination noted a painless wheelbase of the left iliac fossa and a perfectly free and painless left hip joint. Workup findings included inflammatory biological markers with moderate elevation of C-reactive protein at 75 mg/L and erythrocyte sedimentation rate of 44 mm at the 1st hour. Hypereosinophilia was noted at 1.6% of the leucocytes formula. The hydatic serology was negative in the case of our patient. The abdominopelvic ultrasound revealed a cyst of the left psoas muscle looking like a hydatid cyst measuring 11.8 cm × 5.85 cm without other lesions particularly in the liver. An abdominopelvic CT was ordered to better characterize the mass and relations with its neighboring structures (Figure 1); it showed a hydatid cyst measuring 17 × 6.7 × 5.8 cm associated with an infiltration of the neighboring fat with infracentimetric ganglia, arriving in close contact with the small bowel, the left colon, and the left iliac wing. It is in contact with the left external iliac pedicle, the anterior surface of the acetabulum, and the hip joint capsule where we found an articular effusion. On MRI, it was a type II psoas hydatid cyst according to GHARBI classification, which appeared in hyposignal in T1 and T2 containing a central floating membrane. The cyst arrives in contact with the vertebral body of L4-L5 without intramedullary involvement (Figure 2).

Diagnosis is confirmed, and cyst limits and relations are well defined; the patient was proposed for surgery. We used the left pararectal approach with retro-peritoneal passage. The puncture of the contents of the cyst brought back a purulent liquid (infected hydatid cyst) which was completely sucked up and then extraction of the proliferous membrane with realization of a pericystectomy and abundant washing of the residual cavity with physiological saline. Antimicrobial therapy with ceftriaxone and flagyl was started perioperative.

We followed up the patient for 34 months. Immediate postoperative follow-up was simple, and the patient was discharged in the 4^th^ day after surgery. The patient was seen monthly in consultation during three months and then every six months during two years without noticing any abnormalities on clinical examination and ultrasound control.

## 3. Discussion

Hydatidosis is a zoonotic condition caused by the development of the larval form of canine tenia (*Echinococcus granulosus*) in the host's organism. The definitive host of this tenia is dog, whereas the development in humen is accidental by consumption of parasitized viscera, particularly the liver and lungs of the intermediate host (sheep). Hydatic cyst is endemic in the rural and poor population [2]. The patient reported in this case was native from the rural zone of Kalaa Sraghna city, an endemic zone in Morocco which is known by presence of animal hosts of the parasite. In the 10% of cases that escape the pulmonary and hepatic localization, muscular localization represents only 1 to 3% of all localizations of the hydatic cysts [1, 3, 4], even more rarely in psoas muscle where over the last 40 years, only 41 cases have been reported in the literature. The taenia arrives in the portal circulation after crossing the intestinal mucosa. The portal current carries this embryo to the first dam which is the liver; if not via the hepatic veins, the parasite reaches the inferior vena cava, the right heart, and then the lung which constitutes the second dam. When the hexagonal embryo crosses the two dams, it arrives at the great circulation and can be lodged in any localization of the organism. The clinical diagnosis of the hydatid cyst of the psoas is not easy because of the poor orientation elements; it can be revealed by an abdominal mass in the iliac or lumbar location [5], renitent, fixed to the deep plane with often a conservation of the general state [6, 7]. Some cysts may be revealed by complications such as nerve, urinary or vascular compression, or haematogenous surinfection that can lead to severe sepsis [6, 7]. Radiological exploration is an essential step in the diagnosis of the psoas muscle hydatid cyst. Standard radiology may show arcuate calcification of the flanks, erasure of the psoas shadow, or backflow of intestinal gas [8]. Ultrasound is an innocuous first-line test with a diagnostic reliability estimated at 96%. The ultrasound appearance reproduces the 5 stages of the Gharbi classification and reflects the evolutionary stage of the disease. It is superior to CT for identifying the hydatic nature of the cyst, but it is more efficient in the accuracy of its topography and its reports. Magnetic resonance imaging (MRI) is reserved for cases where the diagnosis remains doubtful [9, 10]. It specifies the location of the lesion, defines its adjacent relationships, and points out any associated vertebral extension. Biology tests are mainly based on the hydatid serology that have positive predictive value and confirms the diagnosis if positive, but negative does not eliminate it as in the case of our patient. This examination should preferably be based on two complementary techniques; quantitative (immunofluorescence—ELISA) and qualitative (immunoelectrophoresis and haemagglutination) [6, 10]. Eosinophilia can be found in the hemogram, but it is inconstant [7].

According to the latest recommendations published by the WHO-IWGE about the treatment of liver hydatid disease, the choice of treatment should be based on the characteristics of the cyst, the experience of the medical staff and the availability of drug therapy/surgery, and the possibility of following the patients in the long term; however, no ideal treatment was defined in the recommendations [8].

Surgery remains the first-line treatment for psoas hydatid cyst. Medical treatment based on albendazole or mebendazole remains an alternative for inoperable cases or in massive recurrence in addition to surgery [1, 3, 4, 11, 12] with good outcomes in terms of reducing cyst size and volume [13]. The extraperitoneal surgical approach is preferable to avoid the opening of the peritoneal cavity to eliminate any risk of intraperitoneal hydatid dissemination [7, 14]. In our patient, both surgery with extraperitoneal approach and medical treatment was indicated to ensure radical treatment of the cyst and avoid dissemination or recurrence. The transperitoneal approach, through a median incision is possible and may be useful to treat at the same time the other intraperitoneal lesions associated and in particular hepatic [1, 3, 4, 6, 7, 9, 15, 16]. Radical treatment is based on total cystectomy; however, adhesions to the vasculonervous elements can make this complete resection difficult or even dangerous. The association with vertebral involvement is another contraindication of total cystectomy since, in this case, the parasite behaves maliciously developing between the bone trabeculae without forming an own cystic wall [16]. It is then necessary to be satisfied with a partial cystectomy, keeping the crown of the pericyst near the vascular and nervous elements to avoid their trauma during the dissection. Nevertheless, some precautions for hydatid cyst surgery must be undertaken, particularly the protection of the operative field by surgical drapes soaked with scolicide (hypertonic serum or oxygenated water) and sterilization puncture of the cyst, as the case in our patient.

## 4. Conclusion

The psoas hydatid cyst remains a very rare entity, especially in children, and can simulate a tumor mass. Hence, the interest of an adequate diagnostic procedure based on ultrasound and CT is usually sufficient to make the diagnosis and specify relations with neighboring organs, thus conditioning the therapeutic approach where surgery remains the only radical treatment. However, we recommend association of antiparasitic treatment to avoid dissemination or recurrence of cyst.
